# Sticking your neck out and burying the hatchet: what idioms reveal about embodied simulation

**DOI:** 10.3389/fnhum.2014.00689

**Published:** 2014-09-24

**Authors:** Natalie A. Kacinik

**Affiliations:** Cognitive Neuroscience of Language Lab, Department of Psychology, Brooklyn College and the Graduate Center, City University of New YorkBrooklyn, NY, USA

**Keywords:** idiom, metaphor, embodiment, amodal symbols, perceptual symbols, mirror neurons

## Abstract

Idioms are used in conventional language twice as frequently as metaphors, but most research, particularly recent work on embodiment has focused on the latter. However, idioms have the potential to significantly deepen our understanding of embodiment because their meanings cannot be derived from their component words. To determine whether sensorimotor states could activate idiomatic meaning, participants were instructed to engage in postures/actions reflecting various idioms (e.g., *sticking your neck* out) relative to non-idiomatic control postures/actions while reading and responding to statements designed to assess idiomatic meaning. The results showed that statements were generally more strongly endorsed after idiom embodiment than control conditions, indicating that the meaning of idiomatic expressions may not be as disconnected from perceptual and motor experiences than previously thought. These findings are discussed in terms of the mirror neuron system and the necessity of pluralistic contributions from both sensorimotor and amodal linguistic systems to fully account for the representation and processing of idioms and other figurative expressions.

## Introduction

Across cultures, languages are rich with figurative expressions that frequently occur when people communicate. For instance, English speakers are estimated to utter approximately 10 million novel metaphors and about 20 million idioms over their lifetime (Cooper, 1999). A metaphor involves identifying a connection between otherwise dissimilar conceptual domains (Hoffman, 1984; Lakoff, 1993; Katz, 1996; Gentner et al., 2001), as illustrated in sentences like *Mary's personality is a magnet*, or *Mary's mind was a whirlpool*; whereas idioms are expressions whose meaning cannot be derived from a systematic or literal processing of the component words (Fraser, 1970; Swinney and Cutler, 1979; Libben and Titone, 2008; Vespignani et al., 2010). For example, Mary could be *sticking her neck out* when she sneaks out of her parents' home to attend a late-night party or *sitting on the fence* about who to vote for in the upcoming class election, and the meaning of the individual words does not enable us to understand these idiomatic expressions.

Since the metaphorical link to the origin of their meaning has been lost or is no longer evident, idioms were initially thought to be dead or frozen metaphors (Katz, 1973; Gibbs, 1994; Keysar and Bly, 1999; Jackendoff, 2002; Caillies and Declerq, 2011). Indeed, the similarities and differences in how metaphors and idioms are understood has been the subject of considerable debate (Cacciari, 1993; Katz, 1996; Sanford, 2008; Caillies and Declerq, 2011), particularly since some evidence suggests that idiom comprehension is at least partly based on the activation of underlying conceptual metaphors (Gibbs and O'Brien, 1990; Gibbs et al., 1997; Sanford, 2008; but see Glucksberg et al., 1993; McGlone, 1996; Keysar and Bly, 1999). Research has also shown that idioms and how they are processed can vary according to their transparency and compositionality (Titone and Connine, 1999; Caillies and Butcher, 2007; Libben and Titone, 2008). Although it is no longer correct to consider idioms as dead metaphors (Gibbs, 1993), it is generally accepted that they are indeed a distinct type of figurative expression whose representation and processing differs from that of metaphors (Cacciari, 1993; Glucksberg et al., 1993; Giora and Fein, 1999; Caillies and Declerq, 2011).

Interestingly, even though idioms are used in conventional language twice as frequently as metaphors according to the previously provided estimate, most research has been focused on metaphors, as evidenced by a standard PSYC INFO search yielding over *11 times* more hits for metaphors than idioms. However, despite the fact that metaphors have been studied to a substantially greater extent than idioms, there has still been a considerable amount of research investigating how idioms are represented and processed (e.g., Gibbs, 1986; Gibbs et al., 1989; Hamblin and Gibbs, 1999; Titone and Connine, 1999; Peterson et al., 2001; Tabossi et al., 2005, 2008; Sprenger et al., 2006; Smolka et al., 2007; Schweigert, 2009; Fanari et al., 2010; Holsinger and Kaiser, 2013). As indicated by these references and from the idiom literature in general, the vast majority of studies have been aimed at trying to specify how idiomatic expressions are processed and understood. The major research questions have generally centered around investigating the extent to which idiom comprehension is distinct from or relies on the same lexical, semantic, and syntactic processes involved in the processing of regular literal language (Burt, 1992; Peterson et al., 2001; Tabossi et al., 2005; Vespignani et al., 2010), particularly the degree to which the literal meaning of the component words and of the phrase as whole is potentially activated and processed while the idioms is being understood (Sprenger et al., 2006; Smolka et al., 2007; Rommers et al., 2013).

Other lines of research have focused on identifying the factors or dimensions upon which idioms can vary, such as frequency, familiarity, length, decomposability, transparency, predictability, literality and their effects on how idioms are processed (Titone and Connine, 1994a,b; Libben and Titone, 2008; Tabossi et al., 2008; Skoufaki, 2009; Fanari et al., 2010). However, a review of this literature is really beyond the scope of this paper, since the goal of the present study was to see if it would be possible to activate idiomatic meaning motorically as a result of participants simply engaging in positions or actions like “sticking their neck out,” “sitting on the edge of their seat,” or “burying a hatchet,” without being told or presented with the actual idiomatic expressions themselves. In other words, the purpose of the current study was to investigate the extent to which the representations of some idioms may be embodied and grounded in sensorimotor experiences since prior approaches and research on idioms, as mentioned above, have generally been couched in traditional views of cognition and psycholinguistics, with words and phrases presumed to be relatively abstract, arbitrary, amodal symbols.

This type of standard amodal psycholinguistic approach is also evident in the main theories proposed to explain how idioms are processed. For example, one of the earliest theories of idiom comprehension, the lexical representation hypothesis, suggested that the processing of idioms was similar to the processing of long words whose meaning is simply accessed and retrieved from the lexicon (Swinney and Cutler, 1979). More recent theories, like the decomposition account propose that idiomatic representation varies as a function of compositionality such that some component words carry more idiomatic meaning than others (*pop the question* vs. *kick the bucket*), with highly non-decomposable idioms resulting in faster processing and greater “direct access” to meaning (Gibbs et al., 1989). Alternatively, the configuration approach posits that idioms are represented like any other expression, as a configuration of words that are processed on a word-by-word basis until enough words are configured to retrieve the stored idiomatic string and its meaning (Cacciari and Tabossi, 1988; Cacciari et al., 2007; Tabossi et al., 2009). Since the configuration hypothesis proposes that all idioms are processed like regular literal language until the phrase is identified as an idiom whose meaning is then holistically retrieved, it can thus be considered to represent a hybrid of both decompositional and more unitary non-compositional theories. A variety of hybrid accounts have also been proposed, claiming that the literal meaning and syntactic structure of idioms are always represented and processed to some extent, although they differ in the specific manner by which this occurs (Cutting and Bock, 1997; Titone and Connine, 1999; Sprenger et al., 2006; Caillies and Butcher, 2007; Libben and Titone, 2008).

It is such hybrid accounts that currently seem to have the greatest amount of experimental support (Titone and Connine, 1999; Sprenger et al., 2006; Caillies and Butcher, 2007; Libben and Titone, 2008; Tabossi et al., 2009; Caillies and Declerq, 2011; Holsinger and Kaiser, 2013). However, it should be clear that all of these theories have been focused on the processing of idiomatic expressions, particularly how their non-literal meaning is understood given that its connection to the words in the utterance is generally not clear. Across all of these accounts there is nothing to suggest that the words and phrases in these expressions consist of anything other than standard amodal symbols or lemmas (Cutting and Bock, 1997; Sprenger et al., 2006) with links to their literal and figurative conceptual representations.

In contrast to these types of conventional psycholinguistic theories, there is now considerable evidence that much of our cognitive functioning, conceptual representations, and language processes are fundamentally grounded in our sensorimotor and perceptual experiences (Lakoff and Johnson, 1980, 1999; Barsalou, 1999, 2008; Zwaan and Madden, 2005; Gibbs, 2006b). For example, Stanfield and Zwaan (2001) found that participants processed pictures faster when they had the same orientation implied in a preceding sentence, such that they responded faster to a vertical rather than horizontal picture of a pencil after reading *John put the pencil in the cup*, suggesting that implicit perceptual simulations primed participants to more quickly recognize corresponding spatial orientations. Similarly, Zwaan et al. (2002) found that after reading sentences like *The ranger saw the eagle in the sky*, participants were faster to respond by naming or deciding that spatially congruent pictures (i.e., an eagle with outstretched wings) as opposed to incongruent images like an eagle with folded wings corresponded to a word in the sentence, again showing that participants' comprehension was perceptually biased. A series of studies by Matlock and colleagues has shown that the reading latencies of sentences with implied or “fictive” motion such as *the road runs through the valley* were affected by the speed of motion, type of travel or distance conveyed in a preceding story (Matlock, 2004); and conversely, that fictive motion sentences can influence the subsequent interpretation of an ambiguous sentence like *Next Wednesday's meeting has been moved forward two days* (Matlock et al., 2005), as well the duration and manner of an individual's eye movements (Richardson and Matlock, 2007). More recently, Ansorge et al. (2010) have further demonstrated that such embodied effects can even be obtained with masked subliminal spatial prime words like *high* that can facilitate the processing of related target words like *above*, in addition to affecting the performance of spatially congruent or incongruent responses.

Further support for the embodiment of language comes from research showing that conceptual processing can both affect or be affected by the activity of corresponding perceptual and motor brain regions. For instance, an fMRI study by Hauk et al. (2004) showed that simply reading action words referring to the face, arms, and legs (*e.g., lick, pick, or kick*) resulted in somatotopic motor cortex activation in regions corresponding to the body part responsible for that action. In another study of arm- and leg-related words (*e.g., fold, beat, grasp vs. kick, hike, step*) words, Pulvermüller et al. (2005) used transcranial magnetic stimulation (TMS) to disrupt neural activity in arm areas of the left language-dominant hemisphere and obtained faster responses to leg-related terms, whereas TMS applied to leg areas facilitated responses to arm terms. These and other findings described in a review by Fischer and Zwaan (2008) therefore provide considerable support that language comprehension can be facilitated or hindered by perceptual and motor processes.

Although such findings are convincing, the objection could be raised that the concrete or highly imageable nature of the stimuli biases responses in the direction of embodied effects. Figurative language provides a stronger test for embodiment because even when figurative expressions involve concrete terms their meanings typically refer to abstract concepts divorced from their embodied origins. However, there is now a considerable that at least some types of figurative expressions, particularly metaphors, are also embodied. One of the earliest and most comprehensive proposals along these lines was the *conceptual metaphor theory* (*CMT*) proposed in a seminal book by Lakoff and Johnson (1980), suggesting that metaphors are not simply linguistic phenomena but (1) reflect more general cognitive and experiential aspects of how concepts are represented and processed in the human mind, and (2) represent a new tool for gaining insight into the acquisition of conceptual knowledge, particularly our knowledge and understanding of abstract concepts like GOOD or BAD which become metaphorically represented as being high or low in a spatial sense. The validity of underlying conceptual metaphors like GOOD IS UP has been shown in several experiments demonstrating how different concepts and psychological states (i.e., power, affect, the divine, and even real estate) map onto the vertical axis to demonstrate that UP is indeed generally associated with GOOD (Meier and Robinson, 2004, 2006; Schubert, 2005; Giessner and Schubert, 2007; Meier et al., 2007a,b, 2011).

Other studies have examined the PERSONALITY or FRIENDLINESS is TEMPERATURE metaphor and found that incidental experiences with physical warmth (holding hot vs. iced coffee) induced “warm” judgments about others (e.g., trust) (Williams and Bargh, 2008), that people regulate social warmth with physical warmth (i.e., lonelier people had an increased tendency to take warm baths/showers) (Bargh and Shalev, 2012), and that those who were socially ostracized felt physically colder than those who were not (Zhong and Leonardelli, 2008). A recent investigation by Gibbs (2013) examined the embodiment of the RELATIONSHIPS ARE JOURNEYS metaphor by presenting participants with brief passages describing either a smoothly developing relationship or one with difficulties that are still there and have not been overcome, in either metaphorical or non-metaphorical language. When participants were later asked to walk or imagine themselves walking to a marked spot 40 feet away, those presented with the successful relationship walked longer and further than those given the unsuccessful relationship, but only when written in language conveying a journey metaphor. Lastly, simple metaphoric expressions like *swallow your pride* or *spit out the facts* were found to be understood faster when they were preceded by either the actual or imagined corresponding action relative to mismatching action or no movement control conditions, and these findings were not simply due to lexical-semantic activation or associations (Wilson and Gibbs, 2007). A thorough review and discussion about the embodiment of metaphor is beyond the scope of this paper, but readers may consult the following references (Gibbs, 2006a; Gibbs et al., 2006; Gibbs and Matlock, 2008; Ritchie, 2008; Falck and Gibbs, 2012). Most of the aforementioned work has focused on the manner in which concrete, embodied states facilitate the activation of *pre-existing* conceptual knowledge, but it has also recently been shown that higher-order, abstract and ill-defined *processes* like creativity can be enhanced by embodying metaphors (Slepian et al., 2010; Leung et al., 2012; see Eskine and Kaufman, 2012, for a review).

All of these findings suggest that metaphors are more than linguistic expressions; they are indicative of how embodied experiences influence both the activation and processing of various conceptual representations. However, a potential criticism of this work is that the mappings between the embodied source domains and the abstract target domains in many metaphors can be quite direct and obvious (consider the physical/interpersonal warmth research). In addition, the proponents of metaphor embodiment do not claim that their findings “necessarily generalize to all kinds of metaphorical language… [and that] embodied simulation is necessarily central to all aspects of metaphor comprehension” (Gibbs, 2013, pp. 376–377). This point is potentially important with respect to the embodiment of idioms since they have, by definition, generally lost the metaphorical connection to the origin of their meaning (see the graded account and findings by Desai et al., 2013, mentioned in the next section). Another critical issue regarding the embodiment of language and metaphor is the extent to which the activation of sensorimotor is a really fundamental and obligatory part of conceptual representation and processing, or an epiphenomenal byproduct of contextual priming effects or other underlying amodal mechanisms (Mahon and Caramazza, 2008; Dove, 2009). Putting these issues aside, there is now strong support that the comprehension of at least some metaphors relies on embodied sensorimotor simulations, including the results of some recent neuroimaging studies (Chen et al., 2008; Desai et al., 2011, 2013; Lacey et al., 2012).

Contrary to the considerable behavioral and neuroscientific research on the embodiment of metaphors, very little work has been done to investigate the extent to which idioms may also be embodied. This is likely because in contrast to the perceptually rich and verbally creative quality of metaphors, idioms seem like the hallmark of fixed amodal expressions whose meaning must be explicitly learned, stored, and retrieved from memory. Idioms therefore appear to present a potential challenge for embodied theories of cognition because it seems improbable that the comprehension of idiomatic meaning would involve the activation of perceptual and/or motor regions rather than simply linguistic information. However, due to the considerable amount of evidence demonstrating the embodiment of cognition across various domains such as literal and non-literal language like metaphors, there have been a small but slowly growing number of studies to investigate whether idioms are embodied.

Prior to describing those findings, it is worth considering some earlier work that was not really aimed at investigating the embodiment of idioms *per se*, but consisted of behavioral investigations of the extent to which individuals seem to use mental imagery and underlying conceptual metaphors (that appear to be embodied from the evidence above) in the comprehension of idiomatic meaning. The results of this research have generally been mixed, such that some researchers obtained evidence to support that hypothesis (Gibbs and O'Brien, 1990; Nayak and Gibbs, 1990; Gibbs et al., 1997; Gibbs and Bogdonovich, 1999; Nippold and Duthie, 2003), while others have failed to support the hypothesis (Glucksberg et al., 1993; Cacciari and Glucksberg, 1995; Keysar and Bly, 1995, 1999; McGlone, 1996; Glucksberg and McGlone, 1999; Keysar et al., 2000). Recent efforts to examine the embodiment of idioms have mostly focused on using functional imaging and other neuroscientific techniques to determine whether comprehending idiomatic expressions involves the activity of perceptual and motor areas of the brain.

Similar to the behavioral studies cited above, this research has also produced contradictory results. Specifically, a couple of recent fMRI and MEG studies by Boulenger and colleagues showed that sentences with leg- and arm-related words used in an idiomatic or literal sense (e.g., *He kicked the habit* vs. *He kicked the statue*) each activated somatotopically corresponding areas of motor cortex (although the “leg effect” in the MEG only approached significance) and that the time course of this activation was generally similar and relatively rapid, within 150–250 ms, for both types of stimuli (Boulenger et al., 2009 and Boulenger et al., 2012, respectively). These findings therefore generally support the embodiment of idioms, but it is worth noting that the idiomatic sentences in the latter MEG study produced significantly stronger early activation than literal sentences in language regions like the left temporal pole, Broca's area in the left inferior frontal cortex, and the left dorsolateral prefrontal cortex. Overall, however, the brief latencies and region-specific patterns of activation in these studies suggest that word meaning is recruited from sensoriperceptual systems and that idioms are semantically grounded in the motor system.

Some further but very weak evidence for the embodiment of idioms comes from a couple of recent studies by Desai et al. (2013) and Lauro et al. (2013). Both groups of researchers obtained significant activation in sensory and motor regions for metaphorical sentences like *The congress is grasping the state of affairs* or *Matilde throws her sadness far away* (translated from Italian in Lauro et al., 2013), whereas the results for idiomatic sentences (e.g., *The congress is grasping at straws in the crisis*) only approached significance and showed trends toward the expected effects. In both cases the authors argue for a graded account of embodiment suggesting that as the meaning of an expression becomes increasingly more conventional and abstract, as in the transition from metaphoric to idiomatic meaning, the weaker and less likely it is to activate perceptual and motor brain areas. Even though their results for idioms were non-significant, these researchers claim that their overall findings generally support the embodiment of figurative language.

This is in contrast to several studies that have all failed to provide evidence to support the embodiment of idioms (Aziz-Zadeh et al., 2006; Raposo et al., 2009; Cacciari et al., 2011; Cacciari and Pesciarelli, 2013). For example, the study by Cacciari et al. (2011) involved administering TMS pulses to the leg region of motor cortex and was unable to show significant motor evoked potentials (MEPs) in leg muscles for idiomatic sentences, although MEPs were obtained in response to literal and metaphoric stimuli. Furthermore, both fMRI investigations by Aziz-Zadeh et al. (2006) and Raposo et al. (2009) failed to show significant neural activity in corresponding motor or premotor cortices for expressions like *kick the bucket* and *biting off more than you can chew*. In sum, the findings in both the behavioral and neuroscentific literature are contradictory and the extent to which sensorimotor systems contribute to idiomatic meaning remains unclear. Indeed, given that only Boulenger and colleagues have been able to find significant results in the neural domain thus far (Boulenger et al., 2009, 2012), most of the evidence suggests that idioms are not embodied.

As described and to our knowledge, most of the work regarding the embodiment of idioms and language in general, has involved presenting participants with verbal stimuli to see how they subsequently affect their behavioral responses and activate their perceptual and/or motor brain regions. In contrast, the current study took the relatively novel approach of reversing this design, to examine whether putting individuals into sensorimotor states corresponding to certain idioms would activate their meaning and affect participants' subsequent judgments. If embodying idioms solely by having people engage in the relevant actions without exposure to the actual expressions can activate their meaning, it would suggest that perceptual-motor symbols are a fundamental part of their representation and substantially increase our understanding of how idioms are processed, in addition to providing further evidence for embodiment as the foundation of cognitive and linguistic processing. Since the existing support for the embodiment of idioms was weak and it seemed questionable whether the act of “sticking one's neck out” or “sitting on a fence” could really instantiate the corresponding meanings of taking a risk or being undecided, the study was meant to be an initial exploration of this issue with a limited number of stimuli in a highly plausible experimental context. The whole premise of demonstrating idiomatic embodiment in this paradigm hinges upon participants not being aware that the positions and actions they have to perform represent idioms and potentially recognizing the hypothesis.

Specifically, they were told that the purpose of the study was to investigate how reading comprehension may be affected by different positions and movements. We developed a relatively long story involving a crime and subsequent courtroom drama. The whole narrative was designed to read and flow as one coherent story, but it consisted of four parts that were each written to relate to the meaning of a particular idiom. Each portion of the story was followed by a set of four questions designed to measure the extent to which the idiomatic meaning was activated, which was the dependent variable (DV). With respect to both the stories and questions, the idioms were never actually mentioned and considerable effort was taken to avoid using words and phrases closely associated to the idiomatic expressions and their meanings, to prevent them from being simply activated by verbal means. Further information will be provided in the method section, but the idioms used were: *sticking one's neck out, sitting on the fence, sitting on the edge of one's seat, and burying the hatchet*. They were chosen because (1) they could plausibly be worked into the context of the story, (2) involved sustained positions or actions that could be maintained while participants read portions of the story and responded to the questions, and (3) because pre-testing of potential idioms by 30 students (19 female) similar to those participating in the experiment, indicated that were familiar and understood by at least 70% of those individuals. For each part of the story, participants were assigned to one of three conditions (embodied idiom, embodied control, or normal control), where they either performed the position or action corresponding to the idiom, a different control position or action, or were simply seated in a normal comfortable position, respectively, in a counter-balanced manner. In other words, every participant engaged in each condition at least once across the 4 portions of the story, with each condition occurring an equal number of times across participants.

To our knowledge, the present study thus appears to be the first to investigate the embodiment of idioms by seeing whether having participants simply perform an idiomatic action would be enough to induce their meaning and affect subsequent judgments. It was predicted that embodying the idioms compared to the two types of control conditions, would result in stronger responses to the questions in the direction of the idiomatic meaning. Demonstrating the embodiment of idioms through this relatively novel approach (see Wilson and Gibbs, 2007; Leung et al., 2012, for similar efforts with metaphors), would provide further support that non-literal abstract meanings can still be grounded in sensorimotor experiences, particularly with respect to idioms where the evidence has been mixed.

## Methods

### Participants

The participants consisted of 60 Brooklyn College undergraduates (35 females, 25 males) who participated in this research for course credit. Prior to participating in the experiment, they were asked to complete a survey about their language background(s). This was to ensure that they had sufficient exposure to the English language by 5 years of age, and maximize the likelihood that they would be familiar with the idioms and the ability to read and understand the narrative and questions. Although every participant was exposed to English by the time they were 5 years old, the language history forms indicated that 17 individuals had more familiarity and knowledge of another language in those early years. Since many Brooklyn College students come from recently immigrated families it is almost impossible to find “pure” monolingual native English speakers. In fact, only 18 out of 60 participants reported that they were not exposed to any other language(s) by the time they were 5 years old.

As described above, participants were randomly assigned to one of three conditions for *each part of the story* in a counter-balanced order and mixed design, such that each participant experienced each condition over the course of the study, but were only assigned to one condition for the portion of the story corresponding to a particular idiom. In other words, one participant would have sat normally for the first part of the story (normal control condition), performed the idiomatic action for the next part of the story (embodied idiom condition), engaged in another control action for the third part of the story (embodied control condition), and sat normally again (normal control condition) for the last part of the story. Another participant would have embodied the idiom for the first part of the story, then engaged in a control position, then sat normally, and embodied the idiom again for the final part of the story, and so on, with each condition occurring equally often in all possible orders, and resulting in 20 participants per condition for each portion of the story (i.e., idiom).

### Materials and procedure

As mentioned, the story was written and designed to be administered in four sections, corresponding to different idioms and described in further detail below. Every phase of the story began with a couple of introductory sentences to set the scene, followed by 3–4 substantive paragraphs of roughly similar length, ranging from about 300-400 words. Each portion of the story was followed by a set of 4 questions created to assess the strength of the activation of the idiomatic meaning alluded to in the preceding part of the text, and responded to on a 6-point scale (1 = *strongly disagree*, 6 = *strongly agree*), such that the DV consisted of the mean rating across each set of 4 responses. It is important to note that the actual idioms were not mentioned in any of these materials, and care was taken to avoid using words and phrases strongly related to the idiomatic expressions and their meaning. The complete narrative will be described further momentarily, but an example of one section of the story and corresponding questions is provided in Appendix A/Supplementary Materials 1. The full set of materials can be obtained from the author.

The entire story described a court room drama starting with the suspect's account and written so that it could be interpreted like the suspect had *stuck his neck out* (i.e., taken a risk) by getting into a friend's car which ended up resulting in a robbery and accidental murder. This idiom was embodied by having participants literally sit and stick their necks out while reading that part of the story and responding to the questions. An example of one of the risk-related questions that participants had to answer was “Pat was exercising caution as he let Justin drive oddly silent,” which was reverse coded. The next portion of the story consisted of the prosecuting and defense attorneys' explanations of the murder which showed that both sides had good points and the case was not clear cut, to convey the idiom of *sitting on the fence* (i.e., feeling ambivalent about a decision), which was embodied by participants straddling a height-adjustable sawhorse such that only the tips of their toes abutted the floor, resulting in an unbalanced state. The set of items to which participants had to respond are listed in the Appendix. The third part of the story involved the judge's comments leading-up to the delivery of the jury's verdict, written in relation to the idiom of *sitting on the edge of your seat* (i.e., feeling excited or anxious about an outcome), which was embodied with participants literally sitting on the edge of their seat. An example question was “I am eager to hear what the verdict will be.” Finally, the last part of the narrative dealt with the convict's life and thoughts after the guilty verdict, particularly with respect to his partner in crime to relate to the idiom *burying the hatchet* (i.e., the willingness to forgive). In the embodiment condition, participants were presented with two large catering trays. The left tray contained a small hatchet (with its safety guard on), the right tray was filled with dirt, and participants had to bury the hatchet with dirt using a 1-cup scoop. An example item to which participants responded was “Pat will probably overlook Justin's offense by the time they meet again.”

As explained before, participants were assigned to one of three conditions for each portion of the narrative, either (1) the embodiment conditions described above, (2) a normal control condition where they were comfortably seated in front of desk and read normally, or (3) an embodied control condition that involved engaging in a non-idiomatic position or action while reading the passage and answering the questions. The purpose of this latter embodied control condition was to make sure that the effects obtained were not simply due to participants being slightly distracted or uncomfortable while reading and responding to questions in the embodied idiom condition. For the more “positional,” “sticking one's neck out,” “sitting on the fence,” and “sitting on the edge of one's seat” idiomatic actions, participants in the embodied control conditions stood cross-legged while leaning against a wall. However, since the action of “burying the hatchet” involved moving one's hand to scoop dirt and dump it over a hatchet in a neighboring tray, the embodied control condition involved participants sitting at the same desk, but moving dominoes from one side of the table to the other. These conditions were chosen after testing various options because they were judged to be similar to the embodied idiom conditions in terms of positional awkwardness and motoric action.

Participants were instructed that we were investigating the effects of motor information on text comprehension and that they would be required to perform specific positions/actions while reading a story and responding to questions. While giving participants instructions, it is critically important to note that the experimenter never uttered words associated with the idioms and their meaning, let alone the idiomatic expressions themselves, so that they would not be verbally activated. Instead, participants were asked to basically imitate the same position or action demonstrated by the experimenter while they read each part of the story and answered the corresponding questions. Since the study was designed around one continuous and coherent narrative, the participants were all presented with the sections of the story in the same order, but engaged in different positions or actions for each phase based on the conditions to which they were assigned. After the critical idiom embodiment story task, participants completed a distracter task where they read and underlined passages from a text, followed by a questionnaire to assess their understanding of the target idioms (explaining the meaning of example sentences) and their familiarity with the expressions. The entire study took about 75 min to complete.

### Normative data

A separate group of participants (*N* = 31, 15 females) rated the idioms for decomposability, literality, and transparency on 5-point scales, with 1 = *completely non-X* and 5 = *completely X*^1^. Idioms can vary on these dimension and these variables can affect how idioms are processed (Westbury and Titone, 2011). This information was collected to accurately assess our stimuli on these dimensions and to examine which variables are more or less important in determining *which* idioms are likely to be embodied. The ratings were obtained by embedding our critical items into a larger set of idioms, and the resulting data is presented in Table 1.

**Table 1:** **Normative idiom ratings on relevant dimensions**.

**Idiom**	**Decomposability**	**Literality**	**Transparency**
Sticking your neck out	2.52 (1.00)	3.58 (1.03)	3.68 (1.17)
Sitting on the fence	2.13 (1.11)	3.58 (1.36)	3.19 (1.13)
Sitting on the edge of your seat	2.71 (1.10)	3.39 (1.28)	3.16 (0.86)
Burying the hatchet	2.32 (1.38)	3.23 (1.36)	2.26 (1.00)

*Mean ratings of each dimension with standard deviations in parentheses. Higher values indicate stronger endorsement of each dimension*.

## Results

All participants were included in the analyses because none of them correctly identified the real purpose of the experiment or had to be excluded for other reasons. In addition, post-testing results indicated that all four idioms were understood and familiar to the vast majority of participants (see Table 2). Due to the inherent differences between the idiomatic actions, segments of the story, and subsequent questions, the approach used to analyze the data was to conduct separate One-Way analyses of variance (ANOVAs) across the 3 conditions for each portion of the narrative. Specifically, responses to the 4 questions corresponding to each idiom and phase of the study were averaged into a single score to reflect the activation strength of that idiomatic meaning. One-Way ANOVAs were run for each idiom along with Tukey Honestly Significant Difference (HSD) tests.

**Table 2:** **Percentages of participants who understood and were familiar with the idioms**.

**Idiom**	**Understood (%)**	**Familiarity (%)**
Sticking your neck out	88.3	90
Sitting on the fence	86.7	83.3
Sitting on the edge of your seat	96.7	95
Burying the hatchet	95	83.3

Results revealed that actually “sticking your neck out” significantly increased risk judgments (*M* = 4.49, *SD* = 0.61) relative to the embodied control (*M* = 3.71, *SD* = 0.93) and normal control (*M* = 3.8, *SD* = 0.55) conditions, *F*_(2, 57)_ = 6.981, *p* = 0.002, η^2^_*p*_ = 0.197, with the latter two conditions not significantly differing, *p* = 0.92. “Sitting on the fence” also induced more ambivalent judgments (*M* = 4.45, *SD* = 0.58) relative to the embodied control (*M* = 3.69, *SD* = 0.96) and normal control (*M* = 3.49, *SD* = 0.95) conditions, *F*_(2, 57)_ = 7.174, *p* = 0.002, η^2^_*p*_ = 0.201, with the latter two conditions not significantly differing, *p* = 0.74. Literally “sitting on the edge of your seat” significantly increased judgments of excitement (*M* = 5.16, *SD* = 1.13) relative to the embodied control (*M* = 3.84, *SD* = 1.62) and normal control (*M* = 3.68, *SD* = 1.79) conditions, *F*_(2, 57)_ = 5.640, *p* = 0.006, η^2^_*p*_ = 0.165, with the latter two conditions not significantly differing, *p* = 0.94. However, the effect for “burying the hatchet” was not found to be significant, *F* < 1^2^.

**Figure 1:**
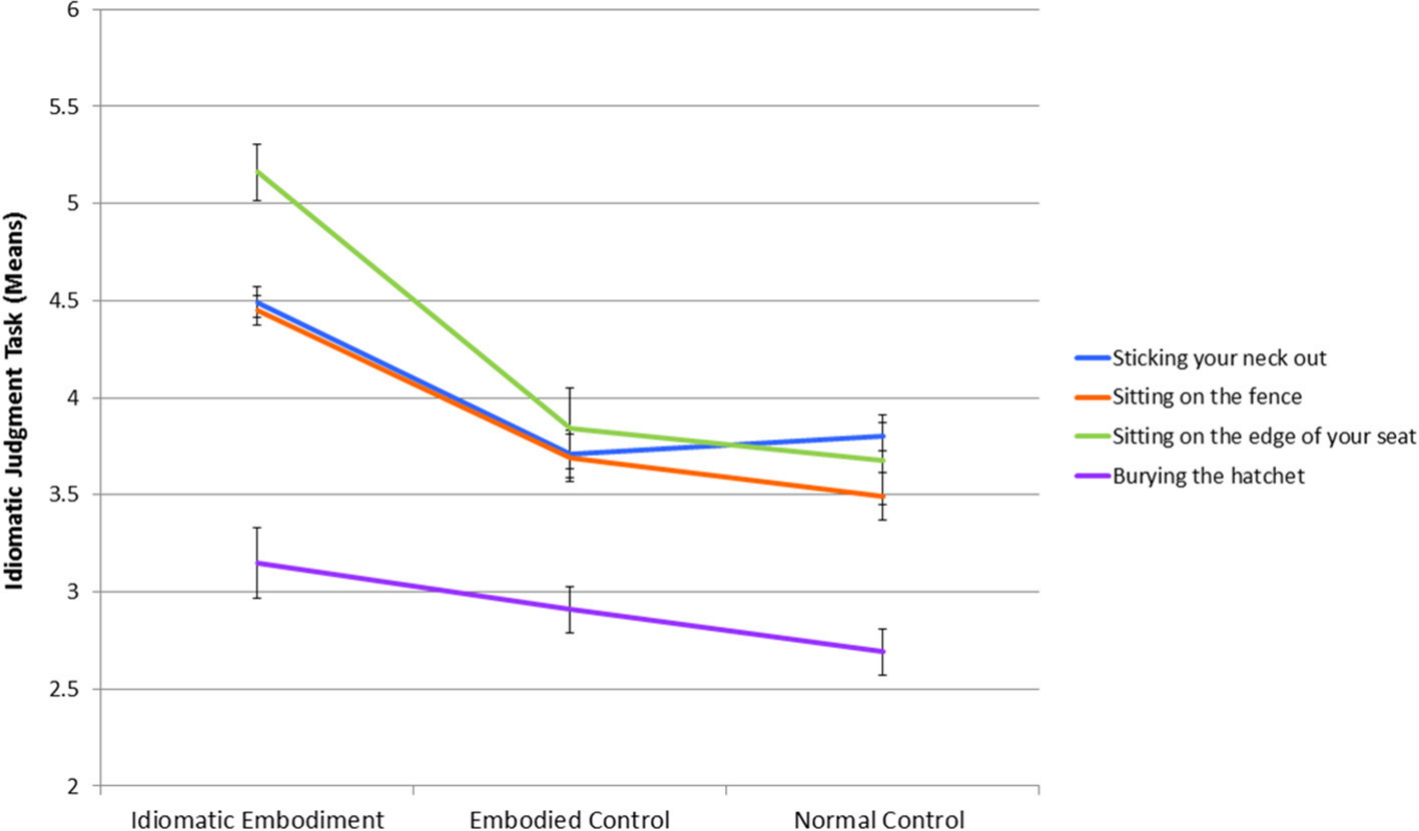
**Mean ratings across the 4 questions designed to assess the strength of the activation of each idiom's meaning (i.e., the idiomatic judgment task)**. Higher numbers indicate a greater endorsement of the idiom's meaning.

These results are displayed in Figure 1, which shows that the idiomatic embodiment condition generally resulted in higher mean responses across the questions designed to measure the activation of each idiom's meaning, than either of the control conditions. Although the analysis for “burying the hatchet” was not significant, Figure 1 shows that the mean ratings followed the same expected pattern. To investigate why, repeated-measures ANOVAs were conducted on the decomposability, literality, and transparency ratings. The idioms were not different in decomposability and literality, *F*s < 1, but did differ in transparency, *F*_(3, 90)_ = 10.489, *p* < 0.001, η^2^_*p*_ = 0.259 (assumptions of sphericity were met). *Post-hoc* pairwise comparisons with Bonferroni corrections showed that *burying the hatchet* was rated significantly less transparent and thus more opaque than the other three idioms, which were not significantly different.

## Discussion

The current research was intended to be an exploratory study about whether embodying idioms could instantiate their meaning to subsequently affect processing and judgments. Recall that previous behavioral and neuroimaging investigations of this issue had produced mixed results (Gibbs and Bogdonovich, 1999; Glucksberg and McGlone, 1999; Keysar et al., 2000; Nippold and Duthie, 2003; Boulenger et al., 2009, 2012; Lauro et al., 2013), with a majority of studies failing to show significant activation in perceptual and motor brain areas in response to idiomatic expressions (Aziz-Zadeh et al., 2006; Raposo et al., 2009; Cacciari et al., 2011; Desai et al., 2013). In contrast to the neuroimaging approach of examining participants' brain activity after exposure to idiomatic stimuli, the present study involved placing participants into the sensorimotor states corresponding to certain idioms to determine whether that could significantly activate their meaning. The results showed that this was indeed the case, thereby providing evidence that at least some idioms have an embodied aspect of their representational structure, and suggesting that embodiment may be more fundamental to the conceptual representations and processing of idiomatic expressions than previously thought.

The approach of the present study is somewhat similar to studies by Ackerman et al. (2010); Leung et al. (2012), and Wilson and Gibbs (2007) that investigated the effects of having participants embody metaphoric actions. Specifically, Ackerman et al. (2010) examined the metaphorical association between physical weight and concepts of severity and importance by showing that participants judged a job candidate to be better if they evaluated him on a heavy vs. light clipboard. Similarly, in another couple of studies investigating the metaphorical links between physically rough textures and harsh or difficult situations, they found that participants judged an ambiguous social situation to be less coordinated (i.e., more difficult and harsh) after working on a puzzle with rough sandpaper-covered pieces compared to those who handled smooth puzzle pieces. The purpose of Ackerman et al.'s experiments was to examine how haptic experiences can affect *interpersonal judgments*. The focus of the study by Leung et al. (2012) was different and designed to investigate whether embodying metaphoric actions is linked to *creative processes*, by seeing if literally thinking about things “on one hand and then the other,” “thinking outside the box,” or “putting 2 and 2 together,” would actually increase measures of creativity. As expected, they found that participants who were seated outside of a box, allowed to walk freely, or who combined the halves of circles together, generally performed better on various convergent and divergent thinking tasks, compared to individuals sitting inside a box, required to walk in a fixed, rectangular path, or who didn't combine circle halves together, respectively.

Finally, in the study by Wilson and Gibbs (2007) participants were trained to perform various actions such as pushing, spitting, and grasping in response to symbols like &,:, and “, displayed on a computer prior to being presented with a phrase like *push the argument, spit out the facts, and grasp a concept*. Wilson and Gibbs found that the responses to those phrases were significantly faster when preceded by matching as opposed to non-matching actions or when preceded by no action. All of these studies thus show that the physical embodiment of metaphors can affect a variety of subsequent processes, but the present research appears to be the first investigation to have taken this approach with idioms. Nevertheless, it must be noted that some of the stimuli from these prior studies consisted of familiar conventionalized phrases like *think outside the box, put 2 and 2 together, swallow your pride, sniff out the truth, and shake off a feeling*, that are likely not that different from the idioms examined in this study. However, in contrast to Wilson and Gibbs (2007) and possibly Leung et al. (2012), participants in the current investigation were never actually presented with the figurative utterances themselves^3^. Wilson and Gibbs provide evidence that their participants were unaware of the connection between the metaphoric actions and expressions, and that their results were not due to lexically-based associations and activation of the figurative meaning. In order to most convincingly demonstrate the embodiment of idioms in the current investigation, we thought it best to completely avoid any sort of linguistic instantiation of the idiom or its meaning.

As mentioned, the goal of this study was to investigate idiomatic embodiment with a small number of idioms in a well-designed and plausible experimental procedure where participants would not recognize that the actions they were asked to do corresponded to idioms and come close to guessing the true purpose of the experiment. Although the pattern of results across the embodied idiom compared to both control conditions clearly supports the embodiment of some idioms, the study is clearly limited by the restricted set of idioms. It will therefore be up to future research to design a study investigating the generalizability of these results to a larger set of stimuli, perhaps with a procedure more akin to that of Wilson and Gibbs (2007), because the length of the story segments and sets of questions from the present experiment resulted in a study that was already over an hour long.

This limitation aside, the present study indicates that the sensorimotor experiences of engaging in idiomatic actions instantiated their meaning to affect participants' processing of the discourse and their responses to the corresponding questions. A potential account of these findings will be presented shortly, but let us first consider other reasons why these results may have been obtained. One possibility is that the current effects could be due to motor imagery rather than embodiment *per se* (Willems et al., 2010; Cacciari et al., 2011; Schuil et al., 2013). Distinguishing these concepts can be difficult and some researchers treat them synonymously, but motor imagery has been defined as the covert or mental “simulation” of bodily movement that involves the activation and monitoring of a motor plan without the overt execution of an action (Willems et al., 2010; Tomasino et al., 2011). Since participants in the current study were actually performing the actions, it seems unlikely and counter to the definition of motor imagery that they would simultaneously be imagining those movements as well. If anything, it's more likely that they may have been imaging the content of the narrative and accompanying questions. A related issue is that the concepts underlying the idioms (e.g., taking a risk, indecision) may have simply been activated as a result of reading the story. While both of the latter phenomena could be true, all of the participants received identical materials (i.e., the same story and questions), so any concepts that could have been imaged or activated were kept constant across participants, but the embodiment conditions still resulted in higher ratings. This indicates that there must be something about the cases where participants performed the idiomatic actions relative to the control conditions that caused them to respond more strongly to the questions. It is also possible that the current findings resulted from a synergistic interaction between the text, questions, and idiomatic actions, rather than just the embodiment of the idioms *per se*. However, even if that was the case, it still means that embodying the idioms contributed something distinct to how the narrative was processed and understood above and beyond the other conditions.

Since participants were never explicitly told or asked about the idioms directly, we also cannot be certain that they were activating the exact intended expressions. There are many idioms conveying risk, some of which would also be compatible with the action of putting one's head and neck forward (e.g., *to put one's head/neck on the block, to put/stick one's head in a noose, put one's neck on the line*). Given human experience about the importance and vulnerability of one's neck, the existence of multiple expressions conveying risk and involving the neck and head is no coincidence and further supports the embodiment of some idioms. Of course there are other idioms like *playing with fire, playing Russian roulette, skating on thin ice, and walking into the lion's den* that also convey risk. It seems unlikely that they would have been activated by the current procedure, but that is an interesting question for future research. Specifically, would activating the concept of risk by the movement of “sticking one's neck out” generalize and facilitate the processing of other “non-neck” idioms like *skating on thin ice*. Similarly, with respect to *being on the fence*, other idioms about indecision also typically convey a similar state of unbalance and sense of going back and forth or side to side (*e.g., hem and haw, go to and fro, be of two minds, and torn/tugged/pulled in 2 directions or between 2 options*). Hence, the extent to which straddling a sawhorse activated *sitting on the fence* rather than one or more of these analogous expressions is unclear, as is the issue of whether other indecision idioms like *being in a quandary, dragging one's feet, or still up in the air*, may have been activated.

Since the current findings indicate that the meaning of certain idioms can be instantiated simply on the basis of sensorimotor experience, we will now try to provide an account for why this may be the case. Specifically, we will propose that one of the main neural mechanisms that could underlie these effects is the human mirror neuron system (HMNS). Mirror neurons were first identified in macaque monkeys as special cells in area F5 [in the inferior frontal gyrus (IFG) and analogous to Broca's area 44 in the human brain], primary and premotor cortex, inferior parietal cortex, and the superior temporal sulcus (Corballis, 2010; Molenberghs et al., 2012; Traxler, 2013). These neurons would fire action potentials both when the monkeys would observe an individual performing a certain action and also when the monkeys engaged in the same action themselves (di Pellegrino et al., 1992; Gallese et al., 1996; Rizzolatti and Craighero, 2004).

Researchers have also identified a similar mirror neuron system in humans, which has been invoked in accounts of language phenomena, particularly the perception of speech, embodiment of semantics, metaphor, interpersonal discourse, and the evolution of language itself, as well as theory of mind, schizophrenia, autism, alexithymia, and multiple sclerosis (Gibbs, 2006a; Gallese, 2008; Corballis, 2010; Molenberghs et al., 2012; Traxler, 2013). However, this research is more controversial and should be considered with caution (Hickok, 2009; Venezia and Hickok, 2009; Arevalo et al., 2012; Molenberghs et al., 2012; Traxler, 2013). The network of regions involved in the HMNS also appears to be very broad, with a recent meta-analysis finding significant levels of activation in 34 Brodmann areas (Molenberghs et al., 2012). However, a generally bilateral set of regions similar to those of the monkeys (i.e., primary motor cortex, ventral premotor cortex, IFG, superior, and inferior parietal lobules) appear to have the strongest support, in addition to the temporal-occipital junction, portions of the limbic system, particularly the amygdala, insula, and cingulate gyrus, and visual, auditory, and somatosensory cortices, depending on the sensory modalities involved (Corballis, 2010; Arevalo et al., 2012; Molenberghs et al., 2012). There has been considerable research and discussion about exactly what the neurons in these brain regions are doing, but the prevailing claim seems to be that they are involved in generating an internal representation, possibly even the “understanding,” of goal-directed actions, rather than just simple imitation (Rizzolatti and Craighero, 2004; Gallese, 2008; but see Hickok, 2009; Corballis, 2010).

We are admittedly hesitant to jump onto the HMNS bandwagon, particularly since the study did not directly investigate this at a neural level. However, the current procedure must have activated the mirror neuron system because participants had to copy the actions demonstrated by the experimenter. This was also true of the control conditions and yet the embodied idiom condition still resulted in significantly greater activation of the idiomatic meaning as measured by the strength of participants' responses to the questions. Therefore, the embodiment of particular idioms is not simply due to the activation of mirror neurons themselves, but rather what those neurons potentially encode with respect to the representation of idioms. Specifically, mirror neurons are important because they interconnect the brain regions involved in the perception of behaviors and the areas responsible for the actual or simulated execution of those actions (Gallese and Lakoff, 2005; Gibbs, 2006a; Fogassi and Ferrari, 2007). As mentioned, the HMNS network is thought to be particularly important for generating an internal representation of an action or behavior and its outcomes or goals (Gibbs, 2006a; Fogassi and Ferrari, 2007; Gallese, 2008). In addition, both the human and monkey research suggests that mirror neurons may be representing actions and their intentions in a more conceptual or cognitive form such that the purpose or consequence of a behavior can be inferred and anticipated (Fogassi and Ferrari, 2007; Gallese, 2008; Corballis, 2010; Traxler, 2013). This possibility combined with the fact that an action does not actually need to be executed but can be encoded into the HMNS by imagination or simulation processes (Gallese and Lakoff, 2005; Wilson and Gibbs, 2007; Gallese, 2008) may explain the embodiment of certain idioms. For instance, the meaning of an idiom like *sticking one's neck out* could become embodied as a result of people encountering individuals being hung or beheaded, and animals being slaughtered by cutting or breaking their necks^4^, in addition to hearing the expression while seeing people put themselves in a variety of risky and dangerous situations, such that these experiences get encoded into an individual's perceptual, motor, and mirror neuron systems.

The idiom of *burying the hatchet* reflects the means by which fighting Native American tribes would end their conflicts and declare peace^5^ (Ammer, 2003). This item is interesting because people (at least those familiar with American history, like the participants in this study) typically know what it means. However, unlike the other idioms in this study, it is the one least likely to be experienced in an occasional movie, show, or book, particularly a visual depiction of the actual procedure, although the expression itself may be encountered more frequently in contexts where forgiveness has or has not occurred. This idiom did not show a significant effect of embodiment, although the results went in the expected direction. In accordance with this finding, “burying the hatchet” was found to be significantly less transparent than the other idioms, indicating that transparency may be a particularly important factor regarding the extent to which an idiomatic expression is embodied. Recall that transparency was defined as the strength or closeness of the connection between the literal and figurative meaning. The current results thus suggest that the weaker and more distant the connection between the literal and figurative meaning, the more likely the processing system needs to rely on amodal linguistic symbols to represent the idiomatic meaning. The present theoretical framework further suggests that transparency really corresponds to the extent to which idioms have been actually physically experienced, either by an individual directly (e.g., someone who shifts forward to the edge of their seat in anticipation of the next scene in a movie) or indirectly through the observation of other individual(s) (e.g., seeing someone shift from side to side while trying to make a decision). In other words, transparency may reflect the strength and frequency with which sensorimotor and mirror neuron systems have been activated by such idiomatic experiences or encounters over time. This would predict that all other things being equal idioms like to *rock the boat* or *muddy the water* should be more embodied due to the fact that most people have likely experienced the instability of being on a boat or water becoming cloudy as dirt or sand is kicked up, compared to expressions like *to have a chip on one's shoulder, shoot the breeze, or paint the town red* which cannot be physically experienced to the same degree. Indeed, it would be interesting to compare more strongly or weakly embodied idioms matched on various other dimensions (e.g., length, frequency, decomposability, transparency, familiarity, and literality) to determine whether a greater degree of embodiment results in idioms that are more easily processed and remembered than less embodied items.

We thus propose that similar to the embodiment established for other aspects of language including metaphors, the meaning of many idioms is grounded in actual or simulated experiences encoded in the HMNS^6^, such that activating the neurons in those corresponding perceptual and motor brain regions can in turn instantiate the idiomatic meaning, as found in the current study. Although it may seem obvious to explain the representation and processing of idioms according to the experiences upon which their meaning is based and understood, prior research has mainly focused on studying the expressions themselves and their properties rather than really considering the situations they describe and the extent to which individuals may have actually directly or indirectly experienced them. The HMNS has been claimed to be one of the fundamental mechanisms responsible for the embodiment of language in general (Barsalou, 2008, 2013; Gallese, 2008; Arevalo et al., 2012; Caligiore and Fischer, 2013; but see Hickok, 2010) with some discussion about how mirror neurons and simulation processes potentially underlie the comprehension of metaphor (Gallese and Lakoff, 2005; Gibbs, 2006a), but to our knowledge the current proposal appears to be first to explicitly suggest that the HMNS may be important for the representation and processing of idioms. A few of the previous neuroimaging studies of idiomatic embodiment have found activity in some of the brain areas involved in the HMNS, most notably the IFG, motor and premotor cortices (Boulenger et al., 2009, 2012; Desai et al., 2013). However, besides suggesting that their results provide support for the notion that abstract figurative meanings are grounded in sensorimotor brain regions, these researchers do not discuss or propose an account of their findings in terms of the mirror neuron system, except for this brief comment by Desai et al. (2013) about the activation obtained for idioms in the IFG (BA44/6) which they describe as being “associated with tool use and thought to be part of the mirror neuron system.” (p. 866).

The current findings in conjunction with some of the neuroimaging research thus appear to be indicative of a bidirectional connection between the meaning of idiomatic expressions and the actual or simulated experiences encoded into individuals' perceptual and motor regions through the HMNS. If this conceptualization is accurate, then encountering an idiomatic expression should re-activate those systems to some degree, but the only way to conclusively confirm this would be by using TMS or studying patients with damage to their mirror neuron systems to see if they show any difficulty with idiom comprehension relative to controls. Since some of the regions identified as being important aspects of the HMNS either overlap or are in close proximity to IFG language areas like BA44 and 45, the most convincing support for the importance of the HMNS would come from showing that impairment of more purely motor or premotor cortices affects idiom processing. Cacciari et al. (2011) appear to have conducted the only study that has really investigated this, but they did not obtain significant MEPs in response to idiomatic sentences after TMS pulses to the leg region of motor cortex. Although this finding fails to support that hypothesis, it should be noted that significant MEPs were shown for metaphorical and fictive motion sentences. Even though those stimuli are figurative, Cacciari et al. note that “the motor component of the verb is preserved” (p. 156) in contrast to idioms where it has vanished. In fact, when individuals were asked to “rate the extent to which the idioms referred to actions… the ratings were extremely low” (communication via review) suggesting that their idiomatic stimuli were generally not considered to be embodied. If preserving the motion component of the verb is critical, as Cacciari et al. have claimed, then this could account for the discrepancy between prior failures to support the embodiment of idioms (Raposo et al., 2009; Cacciari et al., 2011; Cacciari and Pesciarelli, 2013) and the present study which required participants to perform and sustain the idiomatic movements themselves.

Another interesting avenue for future research will be to examine how typically studied features of idiomatic expressions like decomposability and literality relate to the extent of embodiment. Some factors like imageability will undoubtedly be highly correlated with embodiment, but the nature of this relationship for other variables is less clear and should be explored. The linguistic features of idiomatic expressions are an important aspect of their representation and processing, regardless of the degree of embodiment. In addition, since some idioms are most likely either not or very weakly embodied, with their meaning mainly consisting of amodal linguistic symbols (e.g., *something that's the real McCoy, opening a Pandora's box, to get forty winks, kick the bucket, paint the town red, bury the hatchet, or sell someone down the river*), a pluralistic approach like Barsalou et al. (2008) Language and Situated Simulation Theory (LASS) that integrates both linguistic forms and sensorimotor experiences into the human conceptual system may be the most comprehensive and accurate way to account for the wide range of phenomena in natural language, including the variability regarding the embodiment of idioms and other figurative expressions. According to this view, language processing simultaneously triggers the activation of linguistic and sensorimotor simulation systems. The activity of the linguistic system peaks first and is responsible for categorization, spreading activation, and other shallow, word association based processes. The simulation system peaks later and is responsible for deeper conceptual development, which is accomplished through modality-specific simulations, likely involving the HMNS. It is this deeper simulation-based processing that could result in the stronger representations and facilitated processing for more vs. less embodied idioms when other factors remain constant, as suggested earlier.

In conclusion, the present findings show that the process of embodying idioms simply by engaging in the corresponding actions can activate their meaning enough to significantly influence subsequent processing and judgments. This study therefore makes an important contribution to the mixed results in the literature by suggesting that the representation and processing of idiomatic meaning may be more grounded in sensorimotor experiences than previously thought, providing further support for the fundamental importance of embodiment in language comprehension and cognition. Since the current research was limited to a small number of stimuli, it will be up to future studies to investigate a larger and more variable set of idioms to determine the reliability and validity of these results. In spite of this limitation, it is our hope that the relatively novel approach, interesting findings, and proposed account in terms of the HMNS, will stimulate further research along these lines to more thoroughly understand how idioms are represented and processed, particularly with respect to their embodiment.

### Conflict of interest statement

The author declares that the research was conducted in the absence of any commercial or financial relationships that could be construed as a potential conflict of interest.

## Footnotes

^1^Decomposability refers to the extent to which idioms' individual words contribute to their overall figurative meaning. For example, *save your skin* is decomposable because the word “save” relates to the overall idiom meaning (to protect or save yourself). Literality refers to whether the idiom also has a plausible literal meaning. For example, *kick the bucket* figuratively means *to die* and literally means *to strike a pail with your foot*. However, the idiom *under the weather*, which figuratively means to be ill, does not have as clear a literal meaning. Transparency refers to how clearly and directly the figurative idiomatic meaning is related to the expression's literal meaning. While all idioms have a meaningful idiomatic or figurative interpretation, they vary in the degree to which they're transparent or opaque. For example, *jump the gun*, which figuratively means *to start ahead of time*, is relatively transparent because its literal meaning clearly motivates its figurative meaning, whereas the meaning of *kick the bucket* is more opaque because there is no clear and transparent relationship between its literal and figurative meaning.

^2^The same analyses were also conducted on the subset of 43 participants (with the caveat that conditions were no longer fully counterbalanced) after excluding the 17 individuals who had reported greater exposure and knowledge of another language besides English in their first 5 years. These results are reported in Appendix B/Supplementary Material 2 and generally followed the same patterns as the overall analyses. Additional examination of the data on a participant-by-participant basis revealed that the effects were moderately robust across participants, with 65% (or 39/60) individuals showing the expected pattern of stronger ratings in the embodied vs. control conditions, whereas the other 35% (or 21/60 participants) gave ratings in one or both control conditions that were higher or equivalent to the embodied conditions. Interestingly, there was a slight tendency for less native speakers (i.e., those with less exposure to English by 5 years of age) to be less likely to show the expected effects. Specifically, out of the 21 participants not following the expected pattern 9 of them consisted of the less “native” individuals, while only 8 of them followed the predicted pattern. In other words, 31 of the 39 participants who showed the expected results were individuals with more exposure to English in their early years.

^3^With respect to study by Leung et al. (2012), the extent to which creativity metaphors may have been verbally induced while giving participants instructions is unclear. This issue does not appear to be problematic for Ackerman et al. (2010) because their study was not testing specific metaphoric phrases, but rather the underlying conceptual metaphors that give rise to a variety of expressions such as the “gravity of a situation,” “having a rough day,” and someone being “hard-hearted.”

^4^In the past when the idiom originated (see Rogers, 1985; Ammer, 2003) such events would have been experienced in person, but nowadays they are generally encountered in various forms of media.

^5^Another suggestion is that it comes from the expression “hang up one's hatchet” dating back to the early 1300s before the arrival of Christopher Columbus, with the word “bury” replacing “hang up” in the 1700s (Ammer, 2003).

^6^The fact that simulated or imagined actions can still be encoded into the HMNS is particularly critical for explaining how idioms like sticking one's neck out, stabbing someone in the back, or biting someone's head off could still be embodied without individuals having to personally perform those actions. An interesting question for future research would be to examine the degree to which such idioms may be less strongly embodied than idioms like gritting one's teeth or to be left out in the cold that most people have directly experienced.
