# Supplementary material for: Sticking your neck out and burying the hatchet: what idioms reveal about embodied simulation
Source: Front Hum Neurosci. 2014 Sep 24;8:689. doi: 10.3389/fnhum.2014.00689 (PMC4173310; doi:10.3389/fnhum.2014.00689)
Supplement: Supplementary file 1 [file DataSheet1.DOCX]

**Appendix A or Supplementary Material 1**

Example passage and questions testing the idiom *sitting on the fence*

After Pat defends himself, the prosecutor and defense attorney make their statements. The prosecutor remarks first and is followed by the defense.

*Prosecutor*: Ladies and gentleman of the jury, the man before you pulled the trigger that ended the life of Robert, an innocent man and business owner. While the offender tries to appeal to your senses of compassion and sympathy by expressing his remorse and the “unintentional” nature of the crime, do not let your decisions be biased by your emotions.

Many people are unemployed and facing financial difficulties, but that does not grant one permission to rob others. Pat argues that he should not be held fully accountable for Robert’s death since Justin gave him the gun and the robbery was really his idea. Yet, the story is actually quite simple: two men walked into a business with guns and a robbery occurred. As a result, a man is dead. Someone needs to be held accountable, and it was Pat who technically pulled the trigger. I urge you all to remember this while you are deliberating.

*Defense attorney*: Ladies and gentleman of the jury, there is no doubt that Pat pulled the trigger. He admits this and all of the evidence confirms it. However, we need to consider the implications of a murder charge. This man clearly did not intend or find pleasure in the owner’s death. Instead, however, he felt remorse, suffering, and pain. A true murderer does not feel guilt over his actions, and, in these cases, severe sentences (e.g., life imprisonment) are warranted. It is obvious that Pat is filled with guilt over what happened, and I urge you to consider whether he really deserves such a severe conviction.

Pat is a simple, honest man. He served his country and only wanted to make a decent living. Remember his testimony: he was not even aware that Justin was planning to rob that pawn shop and was under the impression that they were job hunting. Justin put that gun in Pat’s hands, and Pat, like a good friend and well-trained soldier, behaved by reacting instinctively. Pat did NOT pull that trigger by himself. Robert’s murder was the result of his own, Justin’s, and Pat’s actions combined; therefore, it is unfair to focus the responsibility entirely on Pat. I argue that you should use empathy and thoughtfulness in making your decision, and that the severity of our charges against Pat must be reduced.

**5. I think the resolution of this case is clear.**

1 2 3 4 5 6

strongly moderately slightly slightly moderately strongly

disagree disagree disagree agree agree agree

**6. I feel pulled towards each side of the case.**

1 2 3 4 5 6

strongly moderately slightly slightly moderately strongly

disagree disagree disagree agree agree agree

**7. The jury should have no difficulty with their deliberations.**

1 2 3 4 5 6

strongly moderately slightly slightly moderately strongly

disagree disagree disagree agree agree agree

**8. I can see both the prosecutor’s and defense attorney’s points of view.**

1 2 3 4 5 6

strongly moderately slightly slightly moderately strongly

disagree disagree disagree agree agree agree

**Appendix B or Supplementary Material 2**

| Table 3 | | | | | | |  | | | |
| --- | --- | --- | --- | --- | --- | --- | --- | --- | --- | --- |
| Means, Standard Deviations, and Results from ANOVAs conducted on subset of 43 “native” English Individuals (i.e., excluding 17 participants with greater exposure and familiarity with another language in their first 5 years) | | | | | | | | | |  |
| Idiom | Embodiment | Embodied Control | | Normal Control | | ANOVA | | |  |  |
| Sticking your neck out | 4.52 (0.64) | 3.43 (0.90) | 3.77 (0.54) | | *F*(2,40)=8.51, *p*<.01 | | |  |  |  |
| Sitting on the fence | 4.45 (0.58) | 3.86 (1.05) | 3.29 (0.99) | | *F*(2,40)=8.05, *p*<.01 | | |  |  |  |
| Sitting on the edge of your seat | 5.33 (0.97) | 4.14 (1.54) | 3.05 (1.77) | | *F*(2,40)=8.92, *p*<.01 | | |  |  |  |
| Burying the hatchet | 3.34 (1.57) | 2.88 (0.88) | 2.77 (0.72) | | *F*(2,40)=1.04, *p*=.36 | | |  |  |  |

*Note.* Mean ratings across the 4 questions assessing the activation of idiomatic meaning with standard deviations in parentheses. Higher values indicate stronger endorsement of the corresponding meaning.
